# Scientific

**Published:** 1889-03

**Authors:** 


### SCIENTIFIC ITEMS.

**Whence the Corona ?**—The solar eclipse of New Year's day has again brought up the question of the nature of the corona. Of the attempted explanations of this phenomenon, the one ascribing it to a diffraction of the sun's light on the edge of the moon seems to have found most favor, though it is not very clear how light thus diffracted can become visible as a halo without falling upon gaseous matter around the moon.

When the igneous mass out of which our satellite evolved was cast off from that of the earth to seek its own orbit, it is hardly to be supposed that it went without its due portion of those elements which, so far as they remained in a gaseous state, would eventually form an atmosphere. But astronomers say there is no evidence of a lunar atmosphere.

Many years ago the German philosopher Schopenhauer argued, from primary premises, that the moon once contained water like the earth and, since it lost its own heat, became covered with a crust of ice, which he thought accounted for the brilliancy of its reflected light. Recent speculations on the moon's constitution have led to the same rational view, so that our satellite may be said to be getting credited at least with the possession of crystalized water.

Now, the congealment of the moon's water implies the disappearance of aqueous vapors, and an atmosphere deprived of such vapors might be expected to escape detection by telescopic search, because the remaining gases, nitrogen and oxygen, would be invisible. But it may be reasonably presumed that these gases would sufficiently reflect the sun's light to be rendered luminous under the favorable conditions of an occultation, and hence likely the corona—revealing a lunar atmosphere.—A. Partz, in *Scientific American.*

**Glass Cloth.**—Mr. Dubus Bonnet of Lille, France, has invented a process of spinning and weaving glass into cloth. The warp is composed of silk, forming the body and ground-work, on which the pattern in glass appears, as effected by the weft. The requisite flexibility of glass thread for manufacturing purposes is to be ascribed to its extreme fineness, as not less than from 50 to 60 of the original strands are required to form one thread of the weft. The process is slow, for no more than a yard of cloth can be produced in 12 hours. The work, however, is extremely beautiful, and comparatively cheap. A French paper commenting on the discovery says : When we figure to ourselves an apartment decorated with cloth of glass, and resplendant with light, we must be convinced that it will equal in brilliancy all that the imagination can conceive and realize ; in a word, the wonders of the enchanted palaces mentioned in the Arabian tales.—*Sci. American.*

**Chinese have no Nerves.**—The North China *Herald* says the quality of "nervelessness" distinguishes the Chinaman from the European. The Chinaman can write all day, work all day, stand in one position all day, weave, beat gold, carve ivory, do infinitely tedious jobs forever and ever, and discover no more signs of weariness and irritation than if he were a machine. This quality appears early in life. There are no restless, naughty boys in China. They are all appalingly good, and will plod away in school without recesses or recreation of any kind. The Chinaman can do without exercise—sport or play seems to him so much waste labor. He can sleep anywhere, amid rattling machinery, deafening uproar, squalling children, and quarreling adults. He can sleep on the ground, on the floor, on a bed, on a chair, in any position. It would be easy to raise in China an army of a million men, nay, of ten millions, tested by competitive examination as to their capacity to go to sleep across three wheelbarrows, head downward like a spider, their mouths wide open and a fly inside.—*Ib.*

**The Sound of Thunder.**—One of the most terse and succinct descriptions of a natural phenomenon is that recently given by M. Hirn, in which he says that the sound which is known as thunder is due simply to the fact that the air traversed by an electric spark, that is, a flash of lightning, is suddenly raised to a very high temperature, and has its volume, moreover, considerably increased. The column of gas thus suddenly heated and expanded is sometimes several miles long, and as the duration of the flash is not even a millionth of a second, it follows that the noise bursts forth at once from the whole column, though for an observer in any one place it commences where the lightning is at the least distance.

In precise terms, according to M. Hirn, the beginning of the thunder clap gives us the minimum distance of the lightning, and length of the thunder clap gives us the length of the column. He also remarks that when a flash of lightning strikes the ground it is not necessarlly from the place struck that the first noise is heard. Again, he points out that a bullet whistles in traversing the air, so that we can to a certain extent follow its flight, the same thing also happening with a falling meteorite just before striking the earth. The noise actually heard has been compared to the sound produced when one tears linen. It is really due to the fact that the air, rapidly pushed on one side in front of the projectile, whether bullet or meteorite, quickly rushes back to fill the gap left in the rear.—*Ib.*

**The Total Solar Eclipse of the Sun, Jan. 1.**—The observation of the parties from the Lick Observatory, at Bartlett Springs, were very complete, and will soon be published by the Observatory. A communication from Prof. J. E. Keeler says:

"My own observations were made with a 6½ in. equatorial telescope, to which was attached a spectroscope with the attachment devised by Hastings, and described in the report of the eclipse at Caroline Island. The phenomena which I observed did not correspond exactly with his observation, but are in partial support of his theory.

Prof. Barnard obtained nine photographs with three cameras equatorially mounted on a polar axis driven by clockwork. His negatives have not been developed yet.

Prof. Hill observed the times of contact, assisted me in my work, and studied the structure of the corona with the finder of the 6½ inch telescope. Time was obtained by telegraph from the Lick Observatory.

Prof. Leuschner obtained seven measures of the light of the corona with a wheel photometer devised and made by Brashear.

Mr. Geo. W. Yount made an oil sketch of the corona, and several other persons made sketches, which were given to the party.

The sky was a little hazy, but all the observations were considered successful."—*Scientific American.*

